# Pomiferin Induces Antiproliferative and Pro-Death Effects in High-Risk Neuroblastoma Cells by Modulating Multiple Cell Death Pathways

**DOI:** 10.3390/ijms26083600

**Published:** 2025-04-11

**Authors:** Manu Gnanamony, Maria Thomas, Thu Hien Nguyen, Korey Brownstein, Pedro A. de Alarcon

**Affiliations:** 1 Department of Pediatrics, University of Illinois College of Medicine Peoria, One Illini Drive, Peoria, IL 61605, USA; tmaria@uic.edu (M.T.); thuhien@uic.edu (T.H.N.); pdealarc@uic.edu (P.A.d.A.); 2 Functional Foods Research Unit, United States Department of Agriculture, Agricultural Research Service, National Center for Agricultural Utilization Research, Peoria, IL 61604, USA; korey.brownstein@usda.gov

**Keywords:** neuroblastoma, cell death pathways, natural compounds, isoflavonoids

## Abstract

Resistance to chemotherapy-induced apoptosis significantly hinders the successful treatment of high-risk neuroblastoma (NB). Natural compounds, such as osajin and pomiferin—isoflavones extracted from Osage orange (*Maclura pomifera* [Raf.] Schneid.)—have known anti-inflammatory and anticancer properties and may have the potential as a therapeutic agent to target conventional drug resistance in NB. In this study, we investigated the antiproliferative and cytotoxic potential of osajin and pomiferin in NB cell lines. Both compounds reduced proliferation and induced cytotoxicity, with pomiferin showing a lower IC50 than osajin. Using multiple techniques, we show that pomiferin induced a dose-dependent increase in apoptosis. In addition to apoptosis, we identified the activation of multiple cell death pathways. Pomiferin induced ferroptosis by inhibiting GPX4 and increasing lipid peroxidation. In addition, pomiferin treatment significantly impaired autophagic machinery. LAN5, a *MYCN*-amplified cell line, showed increased gasdermin E cleavage in response to pomiferin, suggesting pyroptosis. No changes were observed in phosphorylated MLKL, indicating the absence of necroptosis. In conclusion, our comprehensive evaluation demonstrates that pomiferin activates multiple cell death pathways in high-risk NB cells, potentially offering a valuable strategy to overcome drug resistance to conventional chemotherapy.

## 1. Introduction

Neuroblastoma (NB) is the most common extracranial solid tumor occurring in early childhood [1]. Aberrant expression of *MYCN/MYC* is strongly associated with aggressive NB subtypes, driving poor prognosis and the rapid progression of the disease [2,3]. Aggressive cancers often circumvent programmed cell death by exploiting normal cellular processes to evade cell death pathways and support their survival and proliferation [4]. Therefore, exploring drugs that target multiple cell death pathways could be a promising strategy for developing more effective cancer chemotherapy.

Pomiferin and osajin are prenylated isoflavonoids isolated from Osage orange (*Maclura pomifera* [Raf.] Schneid.), a tree native to North America that has antioxidant and anticancerous properties [5,6,7]. Pomiferin in particular has been shown to induce apoptotic cell death in various cancer cell lines [8]. Additionally, it has the capability to trigger autophagic cell death in apoptosis-resistant cancer cells, underscoring its potential to engage multiple cell death pathways [9]. Isopomiferin and its structural analogs selectively inhibit the *MYCN*-amplified subtype of NB by disrupting the *MYCN* transcriptional core module [10]. Thus, pomiferin could be an ideal candidate for targeting NB, particularly for the aggressive and treatment-resistant subtypes.

In this study, we explore the potential of osajin and pomiferin to induce cytotoxicity in in vitro models of high-risk neuroblastoma. Furthermore, we looked at multiple cell death pathways, including apoptosis, ferroptosis, autophagy, pyroptosis, and necroptosis triggered in response to pomiferin.

## 2. Results

### 2.1. Antiproliferative Role of Pomiferin in High-Risk Neuroblastoma

**Figure 1:**
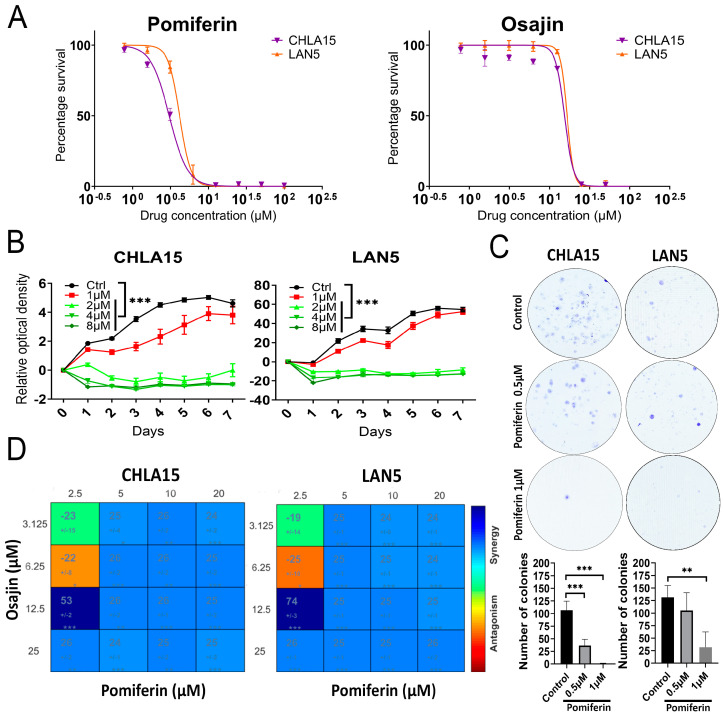
(**A**) Graph showing inhibitory concentration 50 (IC50) of pomiferin and osajin in CHLA15 and LAN5 cell lines measured after 72 h. (**B**) Graph showing proliferation pattern of CHLA15 and LAN5 cell lines treated with increasing concentrations of pomiferin (1–8 µM) as measured by resazurin viability assay for 7 days. *** *p* < 0.001. (**C**) Representative images of colony formation assay showing reduced colonies in CHLA15 and LAN5 cells treated with pomiferin (0.5 and 1 µM) and the corresponding graph showing significant reduction in colony formation in response to pomiferin. ** *p* < 0.01, *** *p* < 0.001. (**D**) Representative plot from Combenefit software showing synergy (blue) between pomiferin (2.5–20 µM) and osajin (3.125–25 µM). * *p* < 0.05, ** *p* < 0.01, *** *p* < 0.001.

In this study, we used two neuroblastoma cell lines: CHLA15 (*MYCN* non-amplified but *MYC*-overexpressing) and LAN5 (*MYCN*-amplified) to investigate the cytotoxic effects of pomiferin and osajin. We performed a resazurin viability assay to assess the impact of osajin and pomiferin on CHLA15 and LAN5 cell lines. Both compounds significantly reduced cell viability in a dose-dependent manner. Osajin had an Inhibitory concentration 50 (IC50) of 14 µM in CHLA15 and 16 µM in LAN5, while pomiferin had an IC50 of 2 µM in CHLA15 and 5 µM in LAN5 (Figure 1A).

Next, we examined the effect of various concentrations of pomiferin on the proliferation of CHLA15 and LAN5 cells. Pomiferin exhibited a dose-dependent inhibitory effect on cell proliferation (*p* < 0.001), with 8 µM causing the maximum inhibition in both cell lines (Figure 1B). We then evaluated the impact of pomiferin (0.5 and 1 µM) on colony formation in CHLA15 and LAN5 cells. There was a dose-dependent decrease in the number of colonies in pomiferin-treated cells compared to dimethyl sulfoxide (DMSO)treated cells (*p* < 0.001) (Figure 1C). To further understand the mechanism behind the anti-proliferative effect of pomiferin, we conducted cell cycle analysis using Hoechst 33342 staining. Pomiferin did not cause cell cycle arrest at any phase in either CHLA15 or LAN5 cells (Figure S1A). This finding is supported by the absence of changes in retinoblastoma (RB) and phospho-RB protein levels in response to pomiferin (Figure S1B). These results suggest that pomiferin treatment induces cell death rather than cell cycle arrest and repair.

Since pomiferin and osajin are often found together in extracts, we looked at the effect of co-treating cells with both compounds and analyzed using Combenefit software version 2.021. We found that pomiferin and osajin synergistically increased cell death in CHLA15 and LAN5 cell lines (Figure 1D).

### 2.2. Pomiferin Triggers Apoptosis in NB

**Figure 2:**
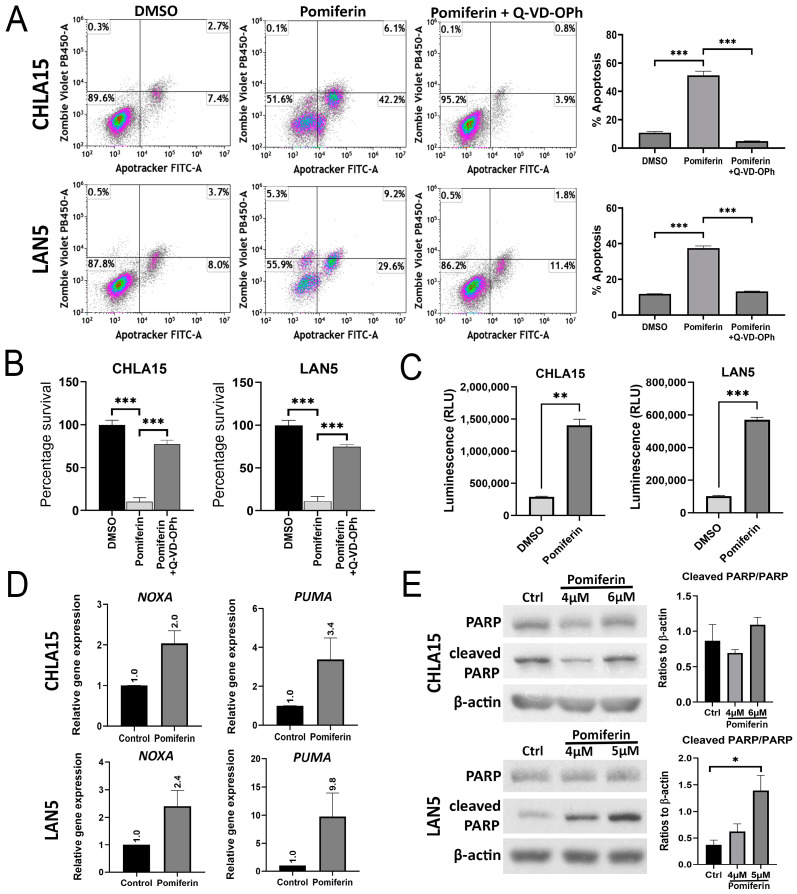
(**A**) Representative Apotracker Green/Zombie Violet plots with quadrant gates of CHLA15 and LAN5 cell lines treated with DMSO, pomiferin (8 µM), and pomiferin (8 µM) co-treated with pan-caspase inhibitor Q-VD-OPh (10 µM) showing viable cell (lower left quadrant), early apoptotic cell (lower right), late apoptotic cell (upper right), and necrotic cell (upper left) quadrants. Corresponding graph shows a significant increase in apoptotic cells in pomiferin-treated cells, which was completely reversed when pre-treated with the Q-VD-OPh (10 µM). *** *p* < 0.001. (**B**) Bar graph showing a reduction in cell viability, measured with resazurin dye, in response to pomiferin (2 µM in CHLA15 and 3 µM in LAN5), which was blocked by Q-VD-OPh (10 µM). *** *p* < 0.001. (**C**) Bar graph showing an increase in luminescence measuring caspase 3/7 activity in response to pomiferin (8 µM) in both CHLA15 and LAN5. ** *p* < 0.01, *** *p* < 0.001. (**D**) Real-time PCR results showing an increase in relative gene expression of pro-apoptotic genes *NOXA* and *PUMA* in response to pomiferin (8 µM). (**E**) Western blot image of PARP and cleaved PARP and corresponding graph showing dose-dependent cleavage of PARP in response to pomiferin (4, 5 µM) in LAN5 but not CHLA15. * *p* < 0.05.

The most common cell death pathway associated with chemotoxic drug therapy is apoptosis. We measured apoptosis using the Apotracker Green/Zombie Violet assay after 24 h of treatment with pomiferin. Pomiferin induced a dose-dependent increase in apoptotic cells in both CHLA15 and LAN5 cell lines (*p* < 0.05, 0.01, 0.001) (Figure S2). Pomiferin-induced apoptosis was reversed by Q-VD-OPh, a pan-caspase inhibitor, as shown by the Apotracker Green flow cytometry assay (*p* < 0.001) (Figure 2A). We observed a similar reversal in cell viability caused by pomiferin when pre-treated with Q-VD-OPh (Figure 2B), suggesting the role of caspases. To explore this further, we performed a caspase 3/7 activity assay and found that pomiferin significantly increased caspase activity in both CHLA15 (*p* < 0.01) and LAN5 (*p* < 0.001) (Figure 2C). Real-time RT PCR analysis showed more than two-fold increase in mRNA levels of the pro-apoptotic genes *NOXA* and *PUMA* (Figure 2D). Western blot analysis showed a dose-dependent increase in cleaved PARP levels in LAN5 (*p* < 0.05) but not in CHLA15 (Figure 2E). Our results strongly indicate that apoptosis is a major pathway of cell death in response to pomiferin.

### 2.3. Ferroptosis Is a Key Modulator of Pomiferin-Induced Cell Death in NB

**Figure 3:**
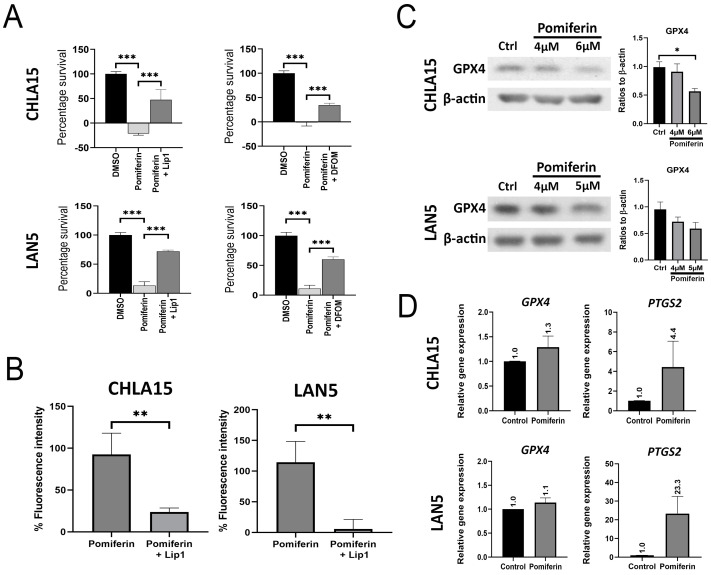
(**A**) Bar graph showing reduced survival in response to pomiferin (2 µM in CHLA15 and 3 µM in LAN5), an effect that was blocked by ferroptosis inhibitor liproxstatin-1 (lip1) (10 µM). *** *p* < 0.001. (**B**) Lipid peroxidation was measured using BODIPY 581/591 C11 staining and flow cytometry after treatment with pomiferin (8 µM). The bar graph shows a significant increase in lipid peroxidation (plotted as percentage fluorescence intensity) in response to pomiferin, which was blocked by liproxstatin-1 (10 µM). ** *p* < 0.01. (**C**) Western blot image showing a reduction in GPX4 expression in response to pomiferin in CHLA15 (4, 6 µM) and LAN5 (4, 5 µM), with a corresponding graph showing a significant reduction in GPX4 levels in CHLA15 but not in LAN5 cell lines. * *p* < 0.05. (**D**) Real-time RT PCR analysis of ferroptosis genes showing a 4.4- and 23.3-fold increase in *PTGS2* mRNA expression in response to pomiferin (8 µM) in CHLA15 and LAN5, respectively.

Cell viability assays showed that cell death induced by pomiferin is blocked by the ferroptosis inhibitor liproxstatin-1 (lip1) and the iron chelator deferoxamine (DFOM), suggesting the involvement of ferroptosis in pomiferin-mediated cell death (*p* < 0.001) (Figure 3A). We measured changes in lipid peroxidation using the BODIPY™ 581/591 C11 staining reagent to study ferroptosis. Pomiferin increased lipid peroxidation in both cell lines, an effect blocked by lip1 (*p* < 0.01) (Figure 3B). We observed a dose-dependent decrease in GPX4 protein levels, a marker of ferroptosis (Figure 3C). Additionally, mRNA levels of *PTGS2*, a known marker of ferroptosis, was significantly elevated severalfold, suggesting ferroptosis. However, we did not observe an increase in FTH1 and DMT1, proteins that are associated with iron import and storage (Figure S3A,B). Our results suggest that ferroptosis is a major pathway in pomiferin-induced cell death, though the exact mechanisms remain to be elucidated.

### 2.4. Changes in Other Cell Death Pathways in Response to Pomiferin in NB

**Figure 4:**
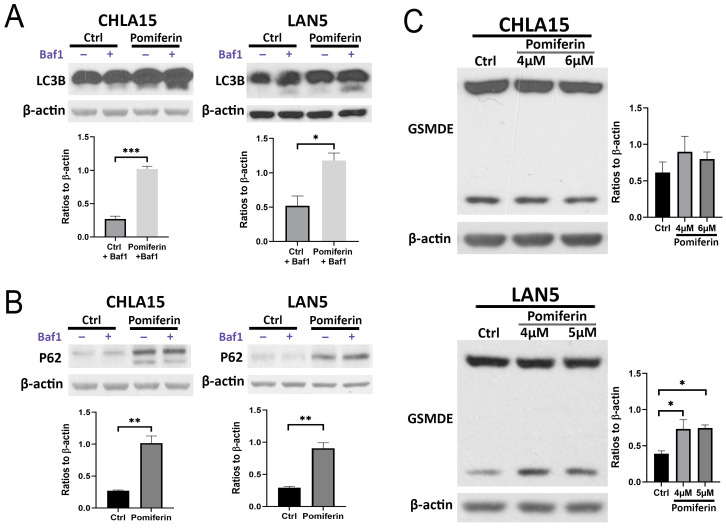
(**A**) Western blot analysis of LC3B in response to pomiferin (8 µM) in the presence and absence of bafilomycin A1 (2 nM) in both CHLA15 and LAN5. Corresponding graph shows significant increase in LC3B-II in pomiferin-treated cells in the presence of bafilomycin A1 showing increased autophagy. * *p* < 0.05, *** *p* < 0.001. (**B**) Western blot images of P62 and the corresponding graph showing an increase in P62 protein levels in response to pomiferin (8 µM) in both CHLA15 and LAN5. ** *p* < 0.01. (**C**) Western blot images of GSDME (full-length and cleaved) and the corresponding graph showing an increase in the cleaved N-GSDME in response to pomiferin (4 and 5 µM) in LAN5 but not CHLA15. * *p* < 0.05.

Changes in autophagy flux were studied by measuring LC3-II turnover in the presence of the inhibitor bafilomycin A1 and P62 in its absence using Western blot. Pomiferin significantly increased LC3-II levels in CHLA15 (*p* < 0.001) and LAN5 (*p* = 0.05), suggesting the activation of autophagy (Figure 4A). Furthermore, pomiferin induced P62 levels in both CHLA15 (*p* < 0.01) and LAN5 (*p* < 0.01) cell lines, suggesting impairment in late-stage autophagy (Figure 4B).

Gasdermin family proteins play a significant role in pyroptosis. We have previously shown that gasdermin E (GSDME) is the highest expressing member of this family in NB cell lines [11]. Therefore, we measured changes in GSDME activation in CHLA15 and LAN5 after pomiferin treatment to assess pyroptosis. In response to pomiferin, N-GSDME increased in LAN5 but not in CHLA15 (Figure 4C). However, we did not observe a difference in *GSDME* mRNA levels upon pomiferin treatment (Figure S4A), suggesting post-transcriptional control.

We then investigated whether necroptosis plays a role in pomiferin-mediated cell death in NB using Western blot analysis of MLKL phosphorylation. We did not find the induction of phospho-MLKL, whereas MLKL levels remained the same in the LAN5 cell line (Figure S4B). In CHLA15, MLKL was not detected even in control samples, suggesting absence or very low expression below the detection limit of the assay.

### 2.5. Pomiferin Synergizes with Conventional Chemotherapy Drugs

**Figure 5:**
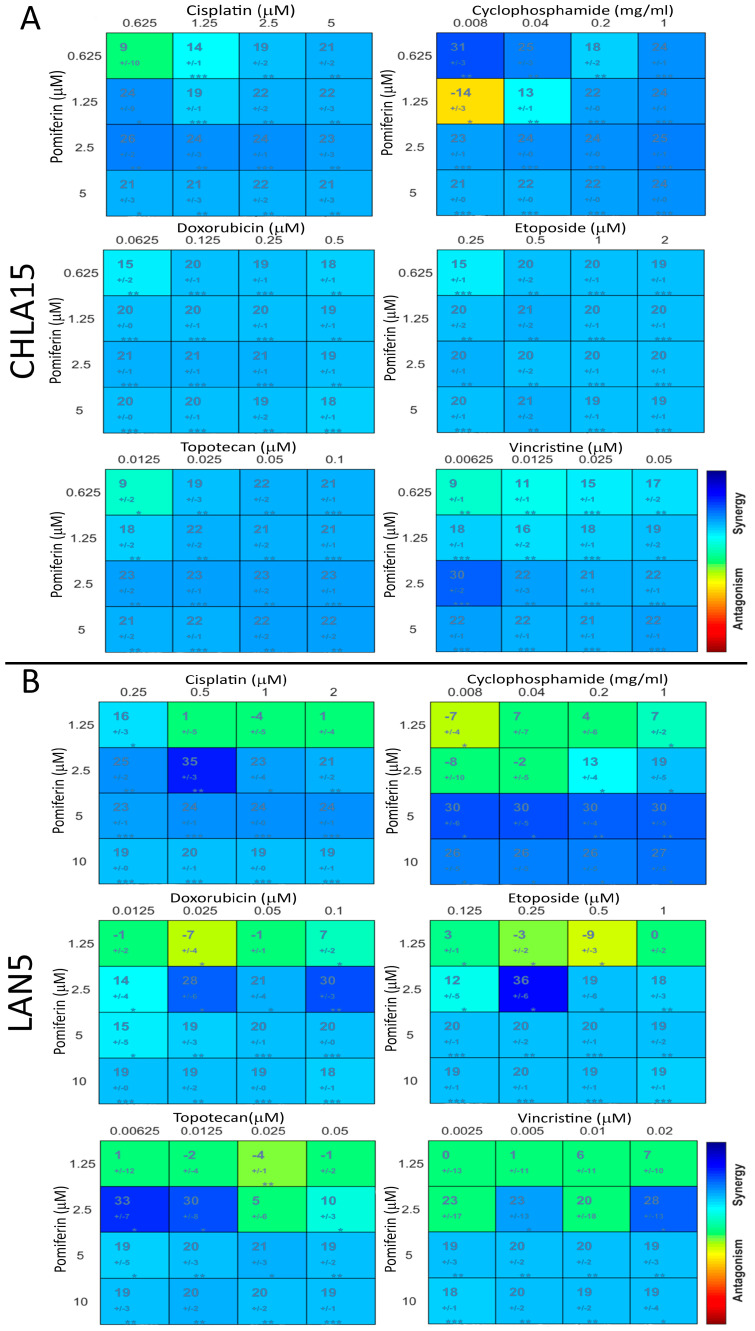
Combenefit software analysis plots showing synergy (blue) between pomiferin (*y*-axis) and six chemotherapy drugs (*x*-axis) in (**A**) CHLA15 (**B**) and LAN5 cell lines. * *p* < 0.05, ** *p* < 0.01, *** *p* < 0.001.

To study the potential benefit of including pomiferin in the current treatment protocol for high-risk NB, we studied the synergy between pomiferin and conventional chemotherapy drugs (cisplatin, cyclophosphamide, doxorubicin, etoposide, topotecan, and vincristine). We found that in both the CHLA15 and LAN5 cell lines, pomiferin showed significant synergy with all six drugs (Figure 5A,B).

## 3. Discussion

The emerging picture of cell death pathways is one of complexity and interdependence. By targeting multiple pathways, more robust and effective treatment strategies can be developed to overcome drug resistance in cancer. Natural products and their derivatives have historically been a significant source of anticancer drugs [12]. In this study, we analyzed the effects of two prenylated isoflavonoids, pomiferin and osajin isolated from Osage orange, on high-risk NB.

In this study, both pomiferin and osajin induced cell death in the micromolar range. Similar IC50 values have been observed in cell lines from other tumors such as kidney, lung, prostate, breast, melanoma, and colon [8,13]. Pomiferin inhibits the growth of glioma cells at 10 µM, whereas in the breast cancer cell line MCF-7, the IC50 is 5.2 µM [7,14]. We did not see a significant alteration to cell cycle phases in response to pomiferin. In support of our results, Yang, R et al. showed that cell cycle regulating genes were not significantly altered in breast cancer cells in response to pomiferin [7]. In contrast, pomiferin mildly increases cells in S phase in ovarian cancer cells [8]. Recent studies in NB show that pomiferin delays tumor growth in mice and inhibits MYCN protein expression in *MYCN*-amplified cell lines, suggesting a potential role for pomiferin in treating NB [10,15]. We found excellent synergy between pomiferin and osajin. These compounds are naturally found together and are often extracted together before separating into individual fractions. This suggests that this synergism occurs because these compounds can interact in distinct yet complementary ways, enhancing their cytotoxic potential in cancer cells.

Using multiple approaches, we show that pomiferin triggers apoptosis in NB cell lines. The activation of apoptosis by pomiferin occurs in other tumor types as well. In a breast cancer cell line, pomiferin increases the expression of apoptotic genes *CANX* and *BCAP31*, though the effect is modest [7]. In ovarian cancer cells, pomiferin increases apoptosis by inducing active caspase 3/7, an effect we have observed as well [8]. Although we observed caspase 3/7 activation in both NB cell lines, PARP cleavage, a common marker of caspase-dependent apoptosis, was found in LAN5 but not CHLA15. Aztopal et al. showed similar results, demonstrating that methanol extracts of *Pelargonium quercetorum* Agnew induce apoptosis without PARP cleavage in non-small-cell lung cancer cell lines [16].

Ferroptosis is an iron-dependent cell death pathway mediated by lipid peroxidation [17]. In this study, pomiferin increased lipid peroxidation in both cell lines, an effect blocked by liproxstatin-1 and DFOM. We also observed a reduction in GPX4, a negative regulator of ferroptosis, suggesting that the action of pomiferin is mediated through GPX4 [18]. GPX4 is vital to protect cells from ferroptosis by reducing dangerous hydroperoxides, therefore a reduction in these levels predisposes cells to ferroptosis [18]. Pomiferin increased the expression of *PTGS2*, which is an mRNA marker of ferroptosis [18]. Our results clearly point to the ferroptosis-activating potential of pomiferin and can be exploited to overcome the resistance to apoptosis. Several natural flavonoids and isoflavonoids have been identified to have either ferroptosis-inducing or -inhibiting properties, depending on the pathology [19]. Dihydroquercetin (DHQ) protects against cigarette-smoke-induced ferroptosis, whereas honokiol induces ferroptosis in acute myeloid leukemia cells [20,21]. To the best of our knowledge, ours is the first study to show a GPX4-mediated pro-ferroptotic role of pomiferin.

Our study clearly points to impaired autophagic pathways in response to pomiferin. In the presence of non-saturating levels of bafilomycin A1, a V-ATPase inhibitor, LC3B-II levels increased, suggesting increased autophagy [22]. We also found an increase in P62 (without bafilomycin treatment), suggesting an impairment in autophagosome degradation. It could be that autophagy impairment predisposes cells to other cell death pathways, such as apoptosis or ferroptosis. An increase in P62 could also indicate the activation of autophagy-independent signaling pathways [23].

Gasdermins are a family of proteins essential for pyroptotic cell death [24]. We have previously shown that in NB cell lines, GSDME is the most expressed gasdermin, and therefore we focused our efforts on this protein for this study [11]. In GSDME-positive cell lines, chemotherapy agents preferably induce pyroptosis instead of apoptosis by cleaving GSDME [25]. Pomiferin treatment resulted in GSDME cleavage in LAN5 but not the CHLA15 cell line in our study. This difference may be attributed to the genetic variation between the cell lines. Specifically, *MYCN*-amplified neuroblastoma has distinct core regulatory mechanisms, which likely result in different pathways controlling cell death and survival [26,27].

Significant interplay exists between regulated cell death pathways. P53, the master regulator of apoptosis and autophagy, also regulates ferroptosis in a context-dependent manner [28]. Both CHLA15 and LAN5 are *P53* wild-type cell lines. A combination of the BTK inhibitor Zanubrutinib and the BCL2 inhibitor navitoclax synergistically induced apoptosis and ferroptosis to suppress growth in double-hit lymphoma [29]. Ferroptosis and necroptosis cooccur in models of kidney damage [30,31]. Ferroptosis and autophagy are also interconnected. Autophagy can induce ferroptosis in some contexts, whereas it can limit damage by ferroptosis in others [32,33]. Therefore, the activation of multiple pathways observed in our study requires careful interpretation and warrants thorough investigations for a comprehensive understanding of the underlying mechanisms in pomiferin-mediated cell death in NB.

For high-risk NB, induction chemotherapy generally involves cycles of cisplatin, cyclophosphamide, doxorubicin, etoposide, topotecan, and vincristine in different combinations [34]. In this study, we found that pomiferin synergizes with all six drugs in both cell lines. Combining our other findings that pomiferin can activate multiple cell death pathways, this result suggests that pomiferin holds significant potential for incorporation into existing treatment regimens.

This is a preliminary study and has several limitations that warrant consideration. Although we have demonstrated the anticancer potential of pomiferin in neuroblastoma cells and its capacity to induce multiple cell death pathways, the underlying upstream biological mechanisms responsible for these effects were not explored. A previous study has shown that pomiferin is a potential inhibitor of histone deacetylase (HDAC) enzyme, which could be one mechanism by which it inhibits cancer cell growth [13]. The inhibition of HDAC activates multiple anti-tumor pathways, including cell cycle arrest, apoptosis, and autophagy [35]. Further investigation is needed to elucidate the specific molecular pathways driving the observed cellular responses to pomiferin in neuroblastoma. Additionally, all experiments in this study were conducted using established cancer cell lines. While these cell lines provide valuable initial insights, they do not fully replicate the complexity of tumor biology in vivo. To better assess the clinical relevance of pomiferin, future studies should include patient-derived cancer cells and in vivo animal models to evaluate the compound′s efficacy, toxicity, and overall tolerability. These investigations are crucial for providing a more comprehensive understanding of pomiferin′s potential as an anticancer agent.

## 4. Materials and Methods

### 4.1. Purification of Osajin and Pomiferin by Flash Chromatography

The procedure for collecting Osage orange fruits and preparing crude extracts had previously been done and is described in Hwang et al. [36]. The crude extract from Osage orange (9.7 g) was dissolved in approximately 150 mL of methanol. The preparative purification was performed on a Büchi Sepacore flash chromatography system controlled by SepacoreRecord chromatography software v 1.4.3000.18063 (Newcastle, DE, USA). The column was a 90 g SiliaSep C18 reverse-phase flash column (230–400 mesh, 40–63 µm, Silicycle Inc., Quebec, QC, Canada). The column was equilibrated with 85% methanol and 15% water containing 0.5% acetic acid at a flow rate of 100 mL/min. After the injection of the samples (15 mL at a time), the column was developed with a linear gradient to 90% methanol over 1 min, held at 90% methanol over the next 10 min, and then a linear gradient to 100% methanol over the next 5 min. The effluent was monitored at 280 nm, and fractions were automatically collected by volume (50 mL). Fractions containing each of the two major absorbance peaks were pooled. The column was washed with methanol between runs and re-equilibrated with 85% methanol. The procedure was repeated until all the extract was used. The two fractions containing the UV-absorbing peaks were pooled and evaporated to remove the methanol and acetic acid, which then allowed the formation of solid yellow crystals in each of the two fractions: pomiferin (first peak) and osajin (second peak). The samples were then evaluated by high-performance liquid chromatography (HPLC).

### 4.2. HPLC Analysis of Osajin and Pomiferin Fractions

To determine the percent concentrations of osajin and pomiferin in the fractions, HPLC analysis was conducted on a Shimadzu LC-20 HPLC system running under Shimadzu LCSolutions version 1.22 chromatography software (Columbia, MD, USA). The column used was an Inertsil ODS-3 reverse-phase C-18 column (5 µm, 250 × 4.6 mm, GL Sciences Inc., Torrance, CA, USA). For osajin and pomiferin analysis, the initial conditions were 30% methanol with 0.25% trifluroacetic acid and 70% water with 0.25% trifluroacetic acid at a flow rate of 1 mL/min. The effluent was monitored at 250 nm. After injection (typically 25 µL), the column was developed to 100% methanol with 0.25% trifluroacetic acid in a linear gradient over 55 min. Five-point standard curves for the evaluation of the concentration of osajin and pomiferin previously purified in this laboratory were used for the determination of extinction coefficients at 250 nm. Quantitation was based on the conversion of the absorbance peak areas to mg/g using the extinction coefficients from the standard curves. The yields were 1.8 g of osajin (>95% pure) and 5.03 g of pomiferin (>95% pure). The recovery was 18% osajin and 53% pomiferin from the starting material, which was a crude extract with 28.6% osajin, 54.1% pomiferin, 1.4% di-hydro-pomiferin, and 1.1% dihydro-osajin, for a total of 86% flavonoids.

### 4.3. Cell Lines, Chemicals, and Media

We acquired CHLA15 and LAN5 cell lines from the Children′s Oncology Group (COG) childhood cancer repository. Both cell lines were maintained in RPMI 1640 medium supplemented with 10% Fetal Bovine Serum and 1% penicillin–streptomycin at 37 °C in a CO_2_ incubator. Periodic mycoplasma testing was done using the Mycoplasma PCR Detection and Elimination Kit (ABM, Richmond, BC, Canada). Purified osajin and pomiferin were dissolved in DMSO as 10 mM stocks and stored as aliquots in −20 °C. For experiments, these compounds were diluted in cell culture media as required. Details of inhibitors and other chemicals used in this study are described in Supplementary Table S1.

### 4.4. Cell Viability Assay

The cell viability was assessed using the Resazurin Cell Viability Assay Kit (Biotium, Fremont, CA, USA). CHLA15 (17,500 cells) and LAN5 (20,000 cells) were seeded in 96-well plates and allowed to adhere for 24 h. Following drug treatment, the plates were incubated for 72 h in a CO_2_ incubator. For the assay, 10 µL of resazurin dye was added to each well, and the plates were incubated for an additional 4 h in the CO_2_ incubator. Absorbance was measured at 450 nm with a correction at 600 nm using an xMark Microplate Spectrophotometer (Bio-Rad, Hercules, CA, USA). Percent viability was calculated using Microsoft Excel for Microsoft 365 (Version 2411). For IC50 determination, cells were treated with 2-fold dilutions of osajin and pomiferin. After 72 h, the cell viability was determined as described above.

### 4.5. Proliferation Assay

CHLA15 (10,000 cells per well) and LAN5 (5000 cells per well) were seeded in 96-well plates 24 h prior to treatment. The cells were then treated with pomiferin (6 replicates) at increasing concentrations from 1 to 8 µM and incubated at 37 °C in a 5% CO_2_ environment. The cell growth was monitored daily for 7 days using the resazurin reagent as previously described. The optical density (OD) readings were measured with colorimetry and normalized to the cell density measured on day 0 and represented graphically as relative OD.

### 4.6. Colony Formation Assay

Cells were counted and treated with pomiferin (0.5 and 1 µM) and then seeded at a density of 2500 cells per well (CHLA15) and 5000 cells per well (LAN5) in 6-well plates. The cells were incubated at 37 °C in a 5% CO_2_ environment. The media were carefully removed and replaced with fresh media every 3 days until colonies became visible. The cells were then washed with phosphate-buffered saline (PBS) and fixed using a 1:7 (*v*/*v*) acetic acid–methanol solution. After fixation, the cells were stained with 0.5% crystal violet for 45 min. The number of colonies was then visually counted.

### 4.7. Synergy Assay

CHLA15 (17,500) and LAN5 (20,000) cells were plated in 96-well plates and grown for 24 h. Cells were then co-treated with increasing doses of pomiferin (in columns) and osajin (in rows). To measure the synergy between pomiferin and chemotherapy drugs, cells were co-treated with pomiferin (in rows) and drugs (in columns). The cell viability was assessed using the resazurin reagent after incubation for 72 h. The optical density values were imported and analyzed using Combenefit software (version 2.021) [37].

### 4.8. Cell Cycle Analysis

CHLA15 (2.5 × 10^6^) and LAN5 (1.5 × 10^6^) cells were seeded and allowed to grow for 24 h before drug treatment. Following a 24 h exposure to the drugs, the cells were collected via trypsinization. They were then stained with Hoechst 33342 at a concentration of 20 μg/mL in growth medium for 25 min at 37 °C, with occasional mixing. The cell cycle phase distribution was analyzed using a CytoFLEX flow cytometer (Beckman Coulter, Indianapolis, IN, USA), and the data were processed with Kaluza software (Beckman Coulter, version 2.2.00001.20183).

### 4.9. Western Blot

The isolation of total cellular protein was performed using the M-PER™ Mammalian Protein Extraction Reagent (Thermo Scientific, Waltham, MA, USA), with the addition of Halt™ protease and phosphatase inhibitors (Thermo Scientific). The protein extracts (30–40 μg per well) were separated on 10% or 12% SDS-PAGE gels and transferred to nitrocellulose membranes (Bio-Rad). The membranes were blocked with 5% nonfat dry milk and incubated overnight with primary antibodies at 4 °C. The membranes were then washed and treated with appropriate secondary antibodies. Bands were visualized using Pierce™ ECL Western Blotting Substrate (Thermo Scientific). Densitometric analysis was conducted using Image J 1.53e. The list of antibodies used in this study is provided in Supplementary Table S2.

### 4.10. Real-Time RT PCR

Total RNA was extracted using Trizol reagent (Invitrogen, Waltham, MA, USA), and its concentration was measured with a Nanodrop 2000 (Thermo Scientific). The cDNA synthesis was performed using the High-Capacity cDNA Reverse Transcription Kit (Applied Biosystems, Waltham, MA, USA), followed by real-time PCR amplification with the PowerUp SYBR Green Master Mix (Applied Biosystems) according to the manufacturer′s cycling conditions. Primer sequences used in this study are listed in Supplementary Table S3. Gene expression levels were normalized to the housekeeping genes *SDHA* and *HPRT* [38].

### 4.11. Apotracker Assay

Cells were collected via trypsinization and washed twice with PBS. After counting, 1 × 10^6^ cells from each treatment were incubated at room temperature for 15 min protected from light with 200 nM Apotracker™ Green (Biolegend, San Diego, CA, USA) and Zombie Violet (Biolegend) diluted 1/500 in 100 μL of cell staining buffer (Biolegend). The cells were then washed twice, re-suspended in 500 μL of cell staining buffer, and analyzed in a CytoFLEX flow cytometer. Dot plots with quadrant gates were created, displaying Apotracker Green fluorescence on the x-axis and Zombie Violet fluorescence on the y-axis. The lower left quadrant indicated healthy cells, the lower right quadrant showed early apoptotic cells, and the upper left and upper right quadrants represented necrotic and late apoptotic cells, respectively. The results are presented as the mean of three different readings. The data were analyzed using Kaluza software.

### 4.12. Caspase 3/7 Assay

CHLA15 (10,000 cells) and LAN5 (15,000 cells) were plated in a white-bottom 96-well plate and incubated in a CO_2_ incubator for 24 h. Drugs were then added and incubated for another 24 h. The plates were removed from the incubator, and an equal volume of Caspase-Glo 3/7 reagent (Promega, Madison, WI, USA) was added and incubated at room temperature for 2 h. Luminescence was measured using a SpectraMax iD3 multi-mode microplate reader (Molecular Devices, San Jose, CA, USA). Media without cells were used as blanks for normalization.

### 4.13. Lipid Peroxidation Assay

Cells were plated at a concentration of 3 × 10^6^ and allowed to grow for 24 h. Following drug treatment, the plates were incubated in a CO_2_ incubator for an additional 24 h. After this, the cells were treated with 2 μM BODIPY™ 581/591 C11 reagent (Thermo Fisher) for 2 h in a CO_2_ incubator. The cells were then collected by trypsinization and washed twice with PBS. The cells were re-suspended in PBS, and fluorescence was measured on a CytoFLEX flow cytometer. The data were analyzed using Kaluza software. For calculations, the mean fluorescence intensity of the FITC/PE ratio was determined and normalized to control.

### 4.14. Statistical Analysis

All statistical analyses were conducted, and graphs were created using GraphPad Prism v10. For all quantifications, experiments were performed in at least three replicates, and the data are presented as mean ± standard deviation unless otherwise noted. Student′s *t*-test was used for comparisons between two groups. Two-way ANOVA with Dunnett post hoc correction was used to analyze proliferation assay. For all multigroup analyses, one-way ANOVA with Dunnett post hoc correction was used. A *p*-value ≤ 0.05 was considered statistically significant.

## 5. Conclusions

The results of this study have confirmed the ability of pomiferin to induce multiple cell death pathways in neuroblastoma cells. In addition to apoptosis, pomiferin triggers ferroptosis, pyroptosis, and autophagy. Our study shows that pomiferin has potential as an anticancer agent. Future studies will (1) delineate the synergistic effects of pomiferin and osajin and (2) study the efficacy of pomiferin in in vivo models of neuroblastoma.

## Data Availability

The data presented in this study are available within the article and the Supplementary Materials.

## Supplementary Materials

The following supporting information can be downloaded at: https://www.mdpi.com/article/10.3390/ijms26083600/s1..
