# Supplementary material for: Pomiferin Induces Antiproliferative and Pro-Death Effects in High-Risk Neuroblastoma Cells by Modulating Multiple Cell Death Pathways
Source: Int J Mol Sci. 2025 Apr 11;26(8):3600. doi: 10.3390/ijms26083600 (PMC12026727; doi:10.3390/ijms26083600)
Supplement: Supplementary file 1 [file ijms-26-03600-s001.zip › Supplemental tables.pdf]

Supplemental table S1: Chemicals used in the study

Name	Manufacturer	Catalog number
QVD-Oph	Medchem express	HY-12305
Liproxstatin-1	Medchem express	HY-12726
Crystal violet	Sigma	C0775-25G
bisBenzimide H 33342 trihydrochloride (Hoechst 33342)	Sigma	B2261-25MG
Dimethyl sulfoxide (DMSO)	Sigma	276855-100ML
Bafilomycin A1,98% (100ug)	Medchem express	HY-100558

Supplemental table S2: Details of antibodies used in the study.

**Name (normal)**	**Name (Full name)**	**Cat no.**	**Manufacturer**
PARP-rabbit	PARP (46D11) Rabbit mAb	9532	Cell Signaling Technology
Cleaved PARP-mouse	Cleaved-PARP (Asp214) (E2T4K) Mouse mAb	32563	Cell Signaling Technology
MLKL-rabbit	MLKL (D2I6N) Rabbit mAb	14993	Cell Signaling Technology
pMLKL-rabbit	Phospho-MLKL (Ser358) (D6H3V) Rabbit mAb	91689	Cell Signaling Technology
P62-rabbit	SQSTM1/p62 (D5E2) Rabbit mAb	8025	Cell Signaling Technology
LC3B-mouse	LC3B (E5Q2K) Mouse mAb	83506	Cell Signaling Technology
GPX4-rabbit	GPX4 Antibody	52455	Cell Signaling Technology
Gasdermin E rabbit	Gasdermin E (E2X7E) Rabbit mAb	19453	Cell Signaling Technology
Rb-mouse	Rb Antibody (IF8)	sc-102	Santa Cruz Biotechnology
pRb-rabbit	Phospho-Rb (Ser780) Antibody	9307	Cell Signaling Technology
b-Actin-mouse	beta Actin Antibody (C4)	sc-47778	Santa Cruz Biotechnology
b-Actin-rabbit	Beta Actin Polyclonal antibody	20536-1-AP	Proteintech
DMT1-rabbit	DMT1/SLC11A2	15083T	Cell Signaling Technology
FTH1-rabbit	FTH1	4393T	Cell Signaling Technology

Supplemental table S3: Primer sequences used in this study.

Name	Sequence (5' to 3')
HPRT-F	TGACACTGGCAAAACAATGCA
HPRT-R	GGTCCTTTTCACCAGCAAGCT
SDHA-F	TGGGAACAAGAGGGCATCTG
SDHA-R	CCACCACTGCATCAAATTCATG
GSDME-F	ACATGCAGGTCGAGGAGAAGT
GSDME-R	TCAATGACACCGTAGGCAATG
NOXA-F	GGTGGAAGTCGAGTGTGCTA
NOXA-R	CCTGAGCAGAAGAGTTTGGA
PUMA-F	GACCTCAACGCACAGTACGAG
PUMA-R	AGGAGTCCCATGATGAGATTGT
GPX4-F	GAGGCAAGACCGAAGTAAACTAC
GPX4-R	CCGAACTGGTTACACGGGAA
PTGS2-F	CTGGCGCTCAGCCATACAG
PTGS2-R	CGCACTTATACTGGTCAAATCCC
