# Supplementary figures and images for: Pomiferin Induces Antiproliferative and Pro-Death Effects in High-Risk Neuroblastoma Cells by Modulating Multiple Cell Death Pathways
Source: Int J Mol Sci. 2025 Apr 11;26(8):3600. doi: 10.3390/ijms26083600 (PMC12026727; doi:10.3390/ijms26083600)

S1A

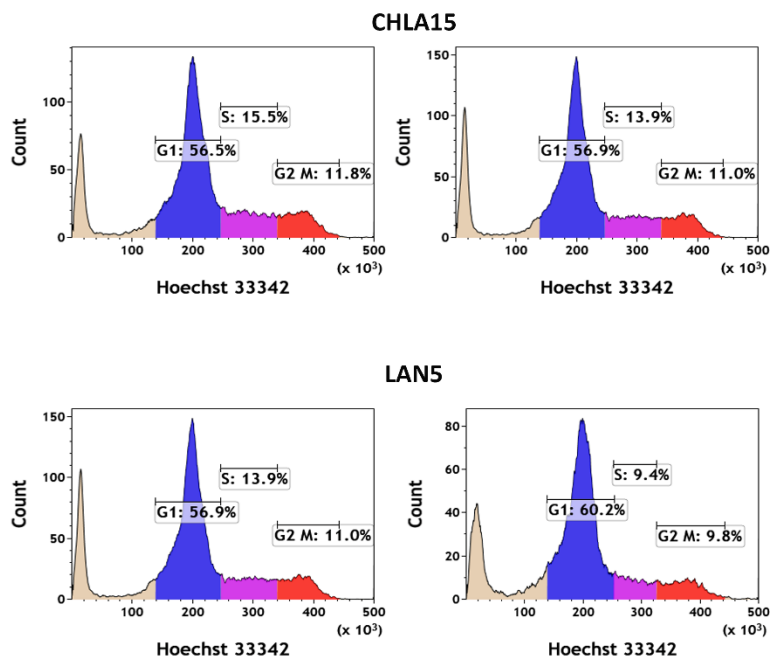

S1B

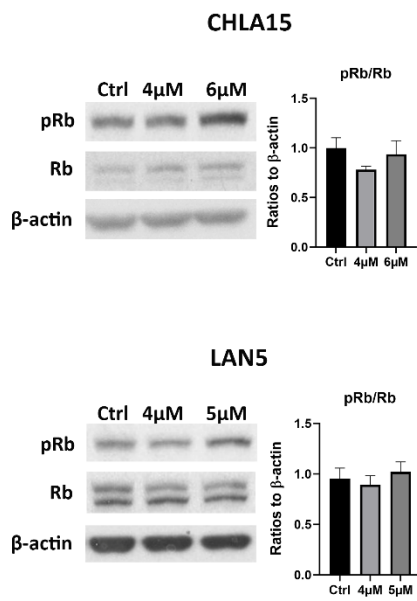

S2

CHLA15

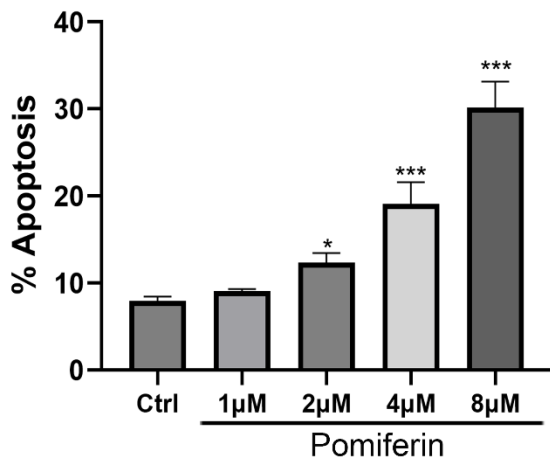

LAN5

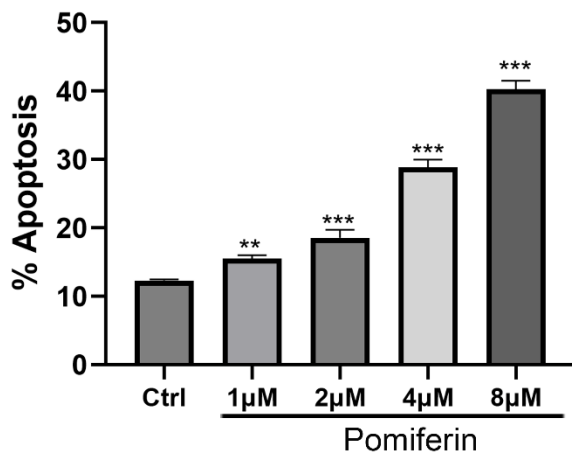

S3A

CHLA15

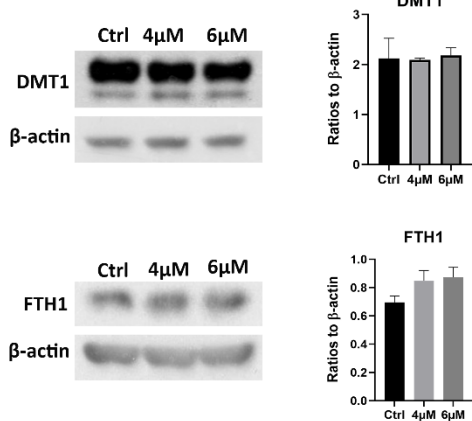

S3B

LAN5

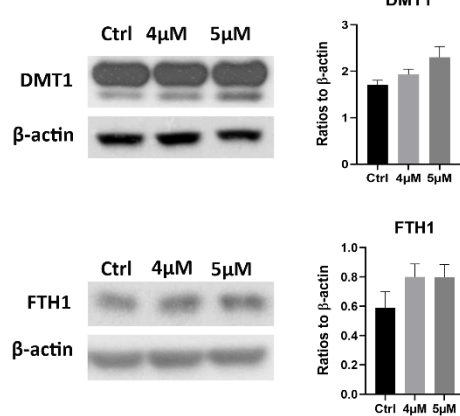

S4A

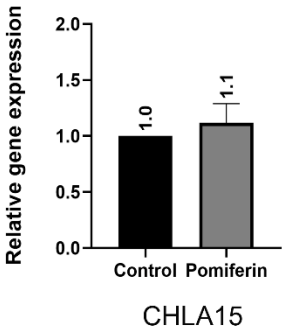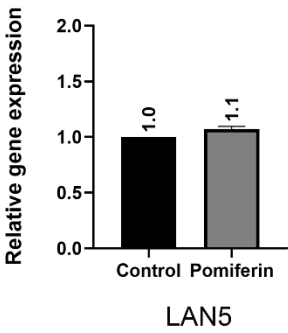

S4B

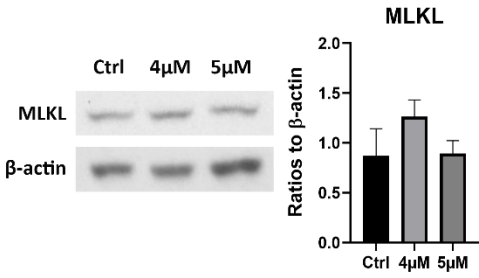

Supplement: Supplementary file 1 [file ijms-26-03600-s001.zip › Supplementary figures.pdf]
